# Babesiosis and Theileriosis in North America

**DOI:** 10.3390/pathogens11020168

**Published:** 2022-01-27

**Authors:** Consuelo Almazán, Ruth C. Scimeca, Mason V. Reichard, Juan Mosqueda

**Affiliations:** 1 Immunology and Vaccines Laboratory, C. A. Facultad de Ciencias Naturales, Universidad Autónoma de Querétaro, Carretera a Chichimequillas, Queretaro 76140, Mexico; joel.mosqueda@uaq.mx; 2 Department of Veterinary Pathobiology, College of Veterinary Medicine, Oklahoma State University, Stillwater, OK 74078, USA; ruth.scimeca@okstate.edu (R.C.S.); mason.reichard@okstate.edu (M.V.R.)

**Keywords:** *Babesia*, *Theileria*, ticks, US, Canada, Mexico

## Abstract

*Babesia* and *Theileria* are apicomplexan parasites that cause established and emerging diseases in humans, domestic and wild animals. These protozoans are transmitted by Ixodid ticks causing babesiosis or theileriosis, both characterized by fever, hemolytic anemia, jaundice, and splenomegaly. In North America (NA), the most common species affecting humans is *B. microti*, which is distributed in the Northeastern and Upper Midwestern United States (US), where the tick vector *Ixodes scapularis* is established. In livestock, *B. bovis* and *B. bigemina* are the most important pathogens causing bovine babesiosis in tropical regions of Mexico. Despite efforts toward eradication of their tick vector, *Rhipicephalus microplus*, *B. bovis* and *B. bigemina* present a constant threat of being reintroduced into the southern US and represent a continuous concern for the US cattle industry. Occasional outbreaks of *T. equi*, and *T. orientalis* have occurred in horses and cattle, respectively, in the US, with significant economic implications for livestock including quarantine, production loss, and euthanasia of infected animals. In addition, a new species, *T. haneyi*, has been recently discovered in horses from the Mexico-US border. Domestic dogs are hosts to at least four species of *Babesia* in NA that may result in clinical disease that ranges from subclinical to acute, severe anemia. Herein we review the pathogenesis, diagnosis, and epidemiology of the most important diseases caused by *Babesia* and *Theileria* to humans, domestic and wild animals in Canada, the US, and Mexico.

## 1. Introduction 

Ticks are obligate hematophagous ectoparasites that negatively impact humans, domestic and wild animals directly through tick bites and blood feeding, and indirectly by transmission of viral, bacterial, and protozoan pathogens [1]. Some of these tick-borne pathogens are responsible for emerging and re-emerging diseases that threaten human and animal health. The increase in tick-borne pathogens and geographical expansion of ticks have been influenced by several factors including global warming, increase in populations of vertebrate reservoir hosts, construction of housing in wooded areas, and increases in human and companion animal outdoor activities in tick-infested areas [2].

Tick-borne pathogens (TBP) are widely distributed in North America (NA), where environmentally diverse conditions favor the development and life cycle of several ticks including *Amblyomma* spp., *Dermacentor* spp., *Haemaphysalis* spp., *Ixodes* spp., and *Rhipicephalus* spp., which infest humans and animals and vector various species of *Babesia* and *Theileria* (Table 1). For example, *I. scapularis*, the black legged tick, which vectors *B. microti* (and *Borrelia burgdorferi* that causes Lyme disease) to humans is widely distributed from the Nearctic regions of Canada and United States (US) southward to the Neotropical regions of Mexico [3]. The ranges of tick vectors and tick-borne diseases (TBD) of humans, domestic and wild animals are increasing or appearing in areas previously considered free, such as Central Canada, where autochthonous cases of babesiosis caused by *B. microti* have been documented [4]. In the US, most of the notifiable infectious diseases annually reported to the Center for Disease Control and Prevention (CDC), are due to pathogens vectored by ticks [5]. According to the CDC, some TBDs such as human babesiosis are doubling in numbers of cases since the disease became notifiable [6] 

Although *B. microti* has been reported in Mexico, significant gaps in the epidemiology of human babesiosis, including definitive identification and distribution of tick vectors, exist [25] and need to be investigated. Additionally, despite the efforts of a national tick control program and targeted research on cattle ticks and TBP of cattle, bovine babesiosis, caused by *B. bigemina* and *B. bovis*, remains one of the biggest obstacles for livestock development in Mexico [12]. Due to the high morbidity and mortality caused by babesiosis to cattle, the transportation and exportation of cattle from infested areas is limited, and the costs of treatment and control represent significant economic losses to the cattle industry [26]. Furthermore, bovine babesiosis represents a continuous concern for naïve cattle in the US, where the tick vector *Rhipicephalus microplus* presents a constant threat of being reintroduced [27].

At least 4 species of *Babesia* are known to infest domestic dogs in NA (Table 1). *Babesia vogeli* is widely distributed from the Central US to Mexico, due to the ubiquitous occurrence of its vector, *R. sanguineus*, the brown dog tick [15]. Cases of canine babesiosis are also caused by *B. gibsoni*, *B. vulpes*, and *B. conradae*, all transmitted through contaminated blood, unknown tick vectors, or iatrogenically [17,28]. In horses, *B. caballi* occurs in Mexico and occasionally in the US, where transmission may be through *Dermacentor* spp. and *Amblyomma* spp. ticks or iatrogenically [18]. In wildlife, *B. odocoilei*, originally identified in white-tailed deer (WTD, *Odocoileus virginianus*), is the causative agent of babesiosis in several free-range and captive ungulates throughout the US and Canada [29] (Table 1). 

At least four species of *Theileria* are known to circulate in horses, cattle, and wildlife in NA (Table 1). *Theileria equi* is prevalent in Mexico and occasional outbreaks occur in the US. Occurrence of *T. equi* in horses has a considerable impact not only on the health of the infected horse but on production as well, as horses or premises may be quarantined and infected horses euthanized to prevent spread and transmission [30,31]. *Theileria haneyi*, a new species, was recently discovered in horses from the Mexico-US border [21]. In cattle, *T. orientalis*, genotype ikeda, has been detected in in beef cattle, associated with introduction of the Asian longhorned tick, *Haemaphysalis longicornis*, into the US [22]. In wildlife, *T. cervi*, which produces a mild infection in WTD, elk, and mule deer, has been documented from the South-Central US to Northern Mexico [32,33]. Herein we review the causative agents, pathogenesis, diagnosis, and epidemiology of babesiosis and theileriosis in humans, domestic and wild animals in NA. 

## 2. *Babesia* species in North America

**Figure 1:**
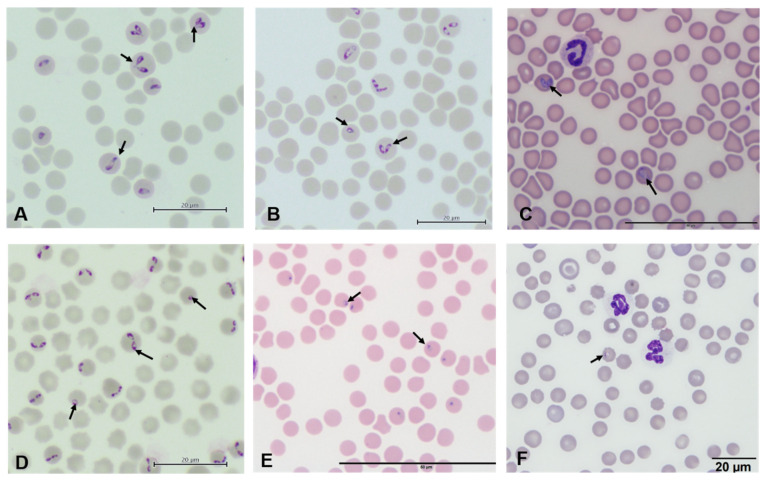
Giemsa-stained red blood cells containing intraerythrocytic inclusions of *Babesia* species (arrows pointed). *Babesia* are generally pear-shaped, arranged individually or in pairs, but other shapes such as rings and ovals may be observed, with measurements of 2.5 to 5.0 µm long in large species (**A**–**C**) and 1.0 to 2.5 µm long in small species (**D**–**F**). (**A**) *B. bigemina*, (**B**) *B. caballi*, (**C**) *B. vogeli*, (**D**). *B*. *bovis*, (**E**) *B. gibsoni*, (**F**) *B. conradae*.

The genus *Babesia* comprises numerous species of protozoans (Table 1) classified within the phylum Apicomplexa, class Piroplasmea, order Piroplasmida, and family Babesidae [34,35]. All species of *Babesia* are transmitted to vertebrate hosts by several species of ixodid ticks. *Babesia* are basophilic, pear-shaped, intraerythrocytic parasites that are distinguished morphologically as either small or large species (Figure 1) [34]. Merozoites measure 1.0 to 2.5 µm in length for small *Babesia* species and 2.5 to 5.0 µm in length for large species [36,37]. Small species identified in NA include *B. microti*, *B. duncani*, *B. gibsoni*, *B. bovis*, *B. odocoilei*, *B. conradae*, and *B. vulpes*. Large species include *B. bigemina*, *B. caballi*, and *B. vogeli*, with one report of the exotic *B. rossi.* In NA, *Babesia* spp. have been reported to affect humans, cattle, horses, dogs, and wildlife including the WTD, elk (*Cervus canadensis*), reindeer (*Rangifer tarandus*), and caribou (*Rangifer tarandus caribou*) [20]. Clinically, babesiosis is characterized by fever, hemolytic anemia, jaundice, and splenomegaly [11,35].

### General Life Cycle of Babesia

The life cycle of *Babesia* spp. involves hard ticks as definitive hosts and vertebrates as intermediate hosts [38]. Development of *Babesia* spp. that occurs in ticks has been described in detail previously [11,13,35]. In vertebrates, infection occurs after passage of *Babesia* spp. sporozoites into host capillaries during tick feeding. The sporozoites penetrate erythrocytes and develop into trophozoites [35]. Trophozoites are round, oval, and ring-shaped entities that multiply asexually by binary fission, forming two to four separate pear-shaped merozoites. Merozoites are released from erythrocytes and infect new erythrocytes [34,35]. *Babesia* spp. merozoites continue invading new erythrocytes until the host dies or the parasite is eliminated. The destruction of erythrocytes in this phase causes hemolytic anemia and hemoglobinuria [13,35]. An immune-mediated response is involved in the pathogenesis and contributes to the disease in naïve hosts [39]. The severity of babesiosis depends on the host’s natural resistance, immune status, acquired resistance, and age [1,11,35]. Some intraerythrocytic forms of *Babesia* spp. become gametocytes under certain stimuli, and are the stage ingested by hard ticks, where sexual reproduction takes place [38]. 

## 3. Human Babesiosis

Human babesiosis is caused by several species of *Babesia* in NA. *B. microti*, a small *Babesia* that is enzootic in the Northeastern US, where the vector, the black legged tick, *Ixodes scapularis* is widely distributed and the transmission cycle is well-established [11]. *B. duncani* causes human babesiosis in the Western US where it is transmitted by the winter tick, *Dermacentor albipictus* [10]. In addition, a few cases of B. divergens-like organisms have been reported in some central states of the US and Washington state [9], and a local case of human babesiosis caused by *B. *microti** was reported in Canada [4]. In addition to ticks, humans become infected with *Babesia* spp. through transfusion of contaminated blood, transplantation of organs with contaminated tissues, and transplacental transmission [11,13,40].

### 3.1. Pathogenesis

The incubation period of *B. microti* in humans varies according to the route of transmission. The prepatent period of *B. microti* is 1 to 4 weeks for tick transmission [41], and 1 to 6 weeks for blood transfusion [11]. Infection of *B. microti* is asymptomatic in 25% of adults and in 50% of children; middle-aged and elderly people are the most adversely affected [6]. Common clinical signs include fever (40.9 °C), fatigue, chills, sweats, anorexia, headache, and myalgia. Less common signs include arthralgia, emotional liability and depression, hyperesthesia, neck stiffness, sore throat, nausea, abdominal pain, vomiting, conjunctival infection, photophobia, weight loss, shortness of breath, and coughing [11].

Lesions caused by *B. microti* include mild to moderate splenomegaly, hepatomegaly, pallor, and/or jaundice. Death may occur, with more frequency in elderly, immunocompromised, or splenectomized patients [8,11]. Other lesions include pharyngeal erythema, jaundice, retinopathy, splinter hemorrhages, and infarcts [13]. Although the course of the disease last two weeks, overall recovery may take up to 18 months. Persistent parasitemia and relapse illness lasts up to 27 months after initial detection and treatment [36]. The severity of human babesiosis depends on immune status and comorbidities. Patients with asplenia, malignancy, HIV, or immunosuppressive drugs experience a severe and long illness [11].

### 3.2. Diagnosis

Identification of patients infected with *Babesia* spp. is determined by detection of piroplasms in blood smears stained with Giemsa or Wright [7]. Detection of *Babesia* spp. DNA using molecular methods such as PCR and qPCR is more sensitive than detection of merozoites in stained blood smears. Serology to detect IgM and IgG antibodies via an indirect fluorescent antibody test (IFAT) is used to differentiate acute and chronic *Babesia* spp. infections [11,24]. The combination of fluorescence in situ hybridization (FISH) and IFAT is useful to detect an active infection and whether the infection is caused by *B. microti* or *B. duncani* [42]. Clinical signs, with a history of the patient visiting tick infested areas or having recently received a blood transfusion, support a babesiosis diagnosis.

### 3.3. Epidemiology

As reviewed by Gray and Herwaldt [40], *B. microti* was identified as the causative agent of human babesiosis in the US in 1969. Currently, confirmed cases of human babesiosis are notifiable to the CDC, and records show that the incidence has increased with about 1000 to 2000 cases per year [6]. Expansion of the geographic range of *I. scapularis*, increases in the numbers of WTD, and construction of houses in natural areas that harbor ticks are factors that influenced this increase. Gray and Herwaldt [40] analyzed the cases of human babesiosis reported to the CDC from 2011 to 2015 in the US. They found 6277 confirmed cases of *B. microti.* The states with the highest number (>90%) of cases were New York, Massachusetts, Connecticut, New Jersey, Rhode Island, Wisconsin, and Minnesota. In all these states, the transmission of *B. microti* by *I. scapularis* is well-established and documented. They also found an increase of human babesiosis cases in Maine and New Hampshire, suggesting an expansion in the foci of transmission. Recently, the information obtained by the CDC from 2011 to 2018 was updated [6]. In total, 14,159 cases of human babesiosis have been reported with an incidence of 5.6 cases per million persons per year. An increasing trend through the years was observed, with most of the reported cases corresponding to the New England and Mid-Atlantic regions, and 50% of them occurred in the states of New York and Massachusetts [6] (Table 2). 

In the Northeast and Upper Midwest US, the white-footed mouse, *Peromyscus leucopus*, is a reservoir of *B. microti.* Larval *I. scapularis* acquire *B. microti* when feeding on infected mice [9,11]. Transovarial transmission of *B. microti* does not occur [43]. Transstadial transmission of *B. microti* occurs as larvae molt to nymphs which may transmit the pathogen to other rodents and humans [9,11]. In endemic locations, most cases of human babesiosis occur in summer, when *I. scapularis* nymphs are most active, with fewer cases in late spring or fall [6,40]. Adult *I. scapularis* preferentially feed on WTD and are not considered as important as nymphs for transmitting *B. microti* to humans. *B. microti* is not infective to WTD, but because deer are key hosts for adult *I. scapularis*, they play a critical role in the epidemiology of human babesiosis by supporting and spreading tick populations [11]. Territorial expansion of *I. scapularis* through the Eastern and Northern US due to deforestation and proliferation of WTD is well-known [2]. As result of the expansion to the north, the first case of human babesiosis by *B. microti* in a child who had no history of traveling outside of the province of Manitoba, Canada, was reported in 2014 [4]. 

*B. duncani* has been identified primarily in the Pacific Northwest of the US and Canada [9]. In the western states of California, Oregon, and Washington, 14 cases of babesiosis due to *B. duncani* were reported 2018 [10]. Adult *D. albipictus* become infected with *B. duncani* by feeding on the infected blood of mule deer, *Odocoileus hemionus*. Infection of *B. duncani* is passed from female *D. albipictus* transovarially to larvae. Larva of *D. albipictus* may transmit *B. duncani* to naïve hosts [10]. Other tick vectors for *B. duncani*, such as *I. scapularis* and *I. pacificus* have been suggested [10]. In addition to *B. microti* and *B. duncani*, few cases of *B. divergens*-like organisms have been reported in the states of Missouri, Kentucky, and Washington, US (Table 2) [44]. In Mexico, *Babesia* spp. was isolated from humans in the region of the Gulf Coast, where babesiosis in domestic animals is enzootic [44]. More recently, *B. microti* was identified as the causative agent of disease in four children from a small community in the southern state of Yucatan [25]. In both cases, the tick vector and reservoirs were not identified. Considerably more research is needed to determine the complete transmission cycle of *Babesia* spp. that cause human babesiosis in Mexico (Table 2).

## 4. Bovine Babesiosis

Bovine babesiosis, also known as piroplasmosis, Texas fever, red water disease, and cattle tick fever, is caused by infection by *B. bovis* and *B. bigemina* [35,45]. *B. bovis* is a small piroplasm and morphologically easy to distinguish from *B. bigemina*, which is a large piroplasm (Figure 1) [37]. In Mexico, *B. bovis* and *B. bigemina* are transmitted by *Rhipicephalus microplus* and *R. annulatus* [13]. Bovine babesiosis may cause mortality in more than 90% of susceptible cattle, which is compounded by the enormous economic losses due to the treatment and control of the ticks. In Mexico, where bovine babesiosis is enzootic, the most recent estimate of economic losses due to control of the disease vector is US $ 573.6 million annually [26]. In the US, *R. microplus* (and thus babesiosis) was eradicated from the country, saving the livestock industry about $3 billion annually [41]. 

**Figure 2:**
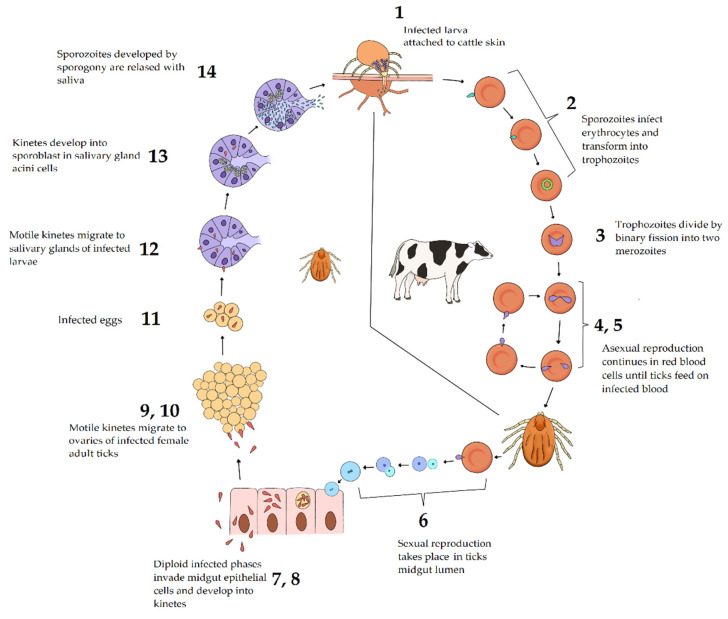
Life cycle of *Babesia bovis*. 1 Infected larva attaches to cattle skin, releasing thousands of sporozoites in host capillaries; 2 *B. bovis* sporozoites invade erythrocytes and develop into ring-shaped trophozoites; 3 two merozoites are generated from each trophozoite by binary fission; 4 mature merozoites, initially joined like two pears at an acute angle, separate before being released from the erythrocyte; 5 some merozoites invade new erythrocytes and develop into trophozoites, while others are taken by adult ticks during blood feeding; 6 sexual reproduction occurs in the invertebrate host, where gametes are released from erythrocytes in the intestinal lumen, and zygote develops after fusion of male and female gametes; 7 an infective phase penetrates the intestinal cells; 8 fission bodies are formed and from them, motile kinetes are developed; 9 kinetes are released from intestinal cells and travel through the hemolymph to different tissues, including ovaries; 10 transovarian transmission occurs when kinetes infect embryos in the ovary; 11 infected eggs are laid by ticks; 12 infected larvae hatch and kinetes migrate to salivary glands acini; 13 kinetes develop into sporoblasts in the salivary gland acini cells; 14 sporogony occurs and sporoblasts develop into sporozoites. Image created with PaintTool SAI, SYSTEMAX 1996–2022.

The life cycles of *B. bovis* (Figure 2) and *B. bigemina* are similar, with both species being transovarially transmitted among *R. microplus*. However, *B. bovis* is transmitted only by larvae as a result of their relatively short feeding times and the biological requirement of *B. bovis* needing only 2 to 3 days after larval attachment for sporozoites to develop. Conversely, development of *B. bigemina* sporozoites requires 9 days, indicating that transmission occurs more readily by *R. microplus* nymphs and adults as a result of the extended feeding times of these stages in comparison to larvae [35]. *B. bigemina* is less pathogenic and remains in the bloodstream for longer than *B. bovis*, resulting in a higher prevalence of *B. bigemina* than *B. bovis* in areas where both pathogens are present [45]. 

### 4.1. Pathogenesis

The incubation periods of *B. bovis* and *B. bigemina* are 6 and 15 days, respectively [40]. During the acute phase of disease, the first clinical sign is fever, and it is during this period that intracellular stages are detected in erythrocytes. Parasitemia in blood smears during the acute phase ranges from 0.1 to 36% [13]. As parasitemia increases, the host’s body temperature rises to 40.0–41.5 °C and persists for 3 days or more. Other signs such as anorexia, hemoglobinemia, hemoglobinuria, jaundice, constipation, dehydration, and abortion associated with high fever in pregnant cattle may appear [35]. As the disease progresses, the animals become lethargic, with muscle wasting, tremors, recumbency, terminal coma, and death [1,35]. *B. bovis* is highly pathogenic, producing neurological signs due to the adherence of infected erythrocytes to endothelial brain capillaries [35]. Although the infection by *B. bovis* causes higher mortality than *B. bigemina*, the latter spreads rapidly, producing severe hemolytic anemia, jaundice, and death [14,35]. 

Gross lesions produced by *Babesia* spp. in cattle include enlargement of spleen and liver, distention of the gall bladder, congestion and dark-colored kidneys, anemia, jaundice, general congestion, petechial hemorrhages, edema in the lungs; and pink coloration of the gray matter of the brain [35].

### 4.2. Diagnosis 

In cattle, the microscopic identification of parasites in stained blood smears is the most useful diagnostic technique due to its low cost, ease, and speed [46]. Although direct identification of *Babesia* spp. in blood smears is useful during acute disease, but not during the chronic phase since the parasitemia is usually low (<1%) and animals are sub clinically infected. For chronic *Babesia* spp. infections, DNA extraction followed by PCR amplification with parasite-specific primers will detect one piroplasm in 10^9^ erythrocytes and differentiate *Babesia* species or isolates [14]. Serological methods including enzyme immunosorbent assays (ELISA) for detection of specific antibodies against *Babesia* spp. in cattle are available and allow screening of a large number of cattle samples for exposure to the parasites [46]. In ticks, identification of *Babesia* spp. kinete stages in hemolymph from *R. microplus* collected in the field can be used to detect the presence of the parasite [14].

### 4.3. Epidemiology

Bovine babesiosis is exotic in Canada and in the US. *R microplus* was eradicated from the US in 1943 but a quarantine zone along the border with Mexico in southern Texas remains to protect against the introduction of ticks imported with livestock or transported by wildlife [13]. Outbreaks of fatal cases of babesiosis in cattle have been recorded in the quarantine zone [47]. More than 1 million head of cattle are imported annually from Mexico to the US, and this economically important trade is at constant risk because of the possibility of incursions of *R. microplus* and babesiosis [12]. Consequently, animal health authorities, stakeholders, regulatory agencies, and tick researchers in Mexico and the US have joined efforts to minimize the risk of reintroduction and re-establishment of *R. microplus* into the US [13,27].

More than 60% of Mexico is infested with the cattle ticks *R. microplus* and *R. annulatus* and considered enzootic for bovine babesiosis [12]. The reported prevalence of *Babesia* spp. in Mexico varies according to the region and detection technique. In the southeast, studies using IFAT and indirect ELISA indicated a seroprevalence of 57.4% and 73.8%, respectively [48,49], while in the northeast, a prevalence of 50.0% for both *B. bovis* and *B. bigemina* was observed [50]. In southern regions, the prevalence of both pathogens can be greater than 90.0% (Table 3) [14,51].

WTD are wild animal hosts for *R. microplus* and *R. annulatus.* Furthermore, free-range WTD and the nilgai (*Boselaphus tromegalus*) are considered key hosts for the dispersion of these ticks in Southern Texas and Northern Mexico [56,65]. Molecular and serological studies indicate that the WTD, nilgai, and the fallow deer (*Dama dama*), carry *B. bovis* and *B. bigemina*, and may be reservoirs of both pathogens (Table 3) [57,58,59,65,66,67,68,69]. It has been demonstrated that WTD cannot become infected with *B. bovis* [66] and other wild ungulates likely play a large role as reservoirs in the epizootiology of bovine babesiosis in Northern Mexico. 

## 5. Canine Babesiosis

In NA, canine babesiosis is caused by *B. gibsoni*, *B. vogeli*, *B. conradae*, and *B. vulpes* [1,17]. *B. gibsoni* and *B. vogeli* are the most common species that affect dogs in the US [15,59]. The presence of *B. vogeli* is more frequently found in southern states where the transmission is due to the brown dog tick, *R. sanguineus* [15]. Although *R. sanguineus* may transmit *B. gibsoni* in other parts of the world, most of the cases due to this protozoon in the US occur by direct blood contact during dog bites in fighting dogs [15]. *B. conradae* was historically found in California [16,28] but has recently been reported in Greyhounds used to hunt coyotes (*Canis latrans*) in Oklahoma [60]. *B. vulpes* has been reported in gray (*Urocyon cinereoargenteus*) and red foxes (*Vulpes vulpes*) from the US and Canada [17] and hunting dogs in the US [36]. Tick vectors of both *B. conradae* and *B. vulpes* have not been identified. 

### 5.1. Pathogenesis

Canine babesiosis affects dogs from all ages, but young animals are more affected [67]. Clinical signs depend on the species of *Babesia*, age of dogs, immune status, and presence of coinfections. All *Babesia* species can cause fever, anemia, and jaundice in dogs [36,68]. Infection with *B. gibsoni* may be subclinical or cause mild to moderate disease [67]. Common clinical signs include apathy, weakness, anorexia, pale mucous membranes, and a poor general condition. The prepatent period of *B. gibsoni* ranges from 14 to 28 days. *B. conradae* is considered highly pathogenic for dogs, producing higher parasitemia than *B. gibsoni* and more pronounced anemia [34]. Predominant lesions produced by canine babesiosis are hemolytic anemia, thrombocytopenia, jaundice, enlargement of lymph nodes, and splenomegaly [67,69]. Anemia is caused by a combination of intravascular and extravascular hemolysis resulting from the parasite-caused injury and rupture of red blood cells due to an increase in the osmotic fragility of the cells [36,69].

### 5.2. Diagnosis

Complete blood cell counts (CBC) in combination with PCR-based techniques are normally performed to establish the diagnosis of canine babesiosis in dogs [28,59]. Microscopical identification of *Babesia* spp. via stained blood smears is commonly used in most laboratories, but has low sensitivity and is not very useful in detecting low parasitemia, especially for small *Babesia* spp. [69]. Nested PCR for detection of 18S rRNA and the internal transcribed spacer (ITS) genes is widely available [16,28,58]. 

### 5.3. Epidemiology

An epidemiological study of canine babesiosis in the US showed that *B. gibsoni* DNA was present in 91% of American pit bull terrier breeds or dogs that have been bitten by another dog [59]. It has been hypothesized that the tick vector of *B. gibsoni* in the US is *R. sanguineus*, but this has yet to be confirmed [15]. *B. vogeli* is distributed in dogs from the Southern US and is frequently found in dogs from kennels that maintain the *R. sanguineus* life cycle [15,59]. *B. conradae* has historically been found mainly in Southern California. Analyses of 55 blood samples of Greyhounds from two kennels in South-Central California found 29 dogs (52.7%) infected with *B conradae* [28]. These infected dogs had no history of tick infestation but had aggressive interactions with coyotes. Analysis of blood samples from 40 dogs originating from four separate kennels in Oklahoma showed that 15 (37.5%) were infected by *B. conradae* [60]. The tick vector and the role of wild carnivores as reservoirs in the epizootiology of *B. conradae* have not been fully determined [60]. Barash et al. [17] investigated the presence of *Babesia* species in 9376 dog samples and found 157 (1.7%) infected with *B. gibsoni*, 19 (0.20%) with *B. vulpis*, and 29 (0.31%) coinfected with *B. gibsoni* and *B. vulpis*. There is one report of *B. rossi* infecting a Boerboel from Texas, but this dog had been recently imported from South Africa where the parasite is enzootic (Table 3) [61]. *B. rossi* is considered an exotic parasite in NA and does not occur naturally in Canada, the US, or Mexico.

Information on canine babesiosis in Mexico is scarce. Detection of *Babesia* spp. in dogs has been performed mostly by microscopical examination of stained blood smears. A large species of *Babesia*, reported as *B. canis*, was isolated for the first time from a natural infected dog from Veracruz [44]. *B. canis* occurs naturally in dogs in Europe and is not known, nor has it previously been reported in dogs from NA. Subsequent study must occur to verify the identification of the large *Babesia* spp. in the dog from Veracruz. In another study performed in the southern state of Yucatan, 4 out of 102 blood smears were positive for *Babesia* spp. [52]. More recently, *B. vogeli* was identified using molecular techniques in *R. sanguineus* and blood collected from dogs in the central state of Morelos (Table 3) [58]. Epidemiological studies have not been conducted in dogs recently, due in part to the lack of serological diagnostic methods, and the status of canine babesiosis remains largely unknown.

## 6. Babesiosis in Cervids

Babesiosis in cervids in NA is caused by B. odocoilei, a small *Babesia* found as singlets, paired pyriforms, or tetrads in the periphery of erythrocytes of cervids [29]. This organism was originally described in the WTD [70], where it was found causing a fulminant, hemolytic disease in splenectomized deer, and emaciation and anemia in intact deer. More recently, *B. odocoilei* was found affecting other ungulates such as elk, reindeer, and caribou [20]. *B. odocoilei* is transmitted by I. scapularis.

### 6.1. Pathogenesis

Lesions caused by *B. odocoilei* in WTD include icterus in the mucous membranes, lungs, brain, and intestinal serosa, and petechial and ecchymotic hemorrhages in the subepicardium [70]. Elk with hyperacute babesiosis present with jaundice, hematochezia, and pigmenturia and immunocompromised animals develop acute hemolytic crisis, with lethargy, pyrexia, icterus, hemoglobinuria, and death [20].

### 6.2. Diagnosis 

Clinical signs in immunocompromised animals and a decrease in packed cell volume are helpful in establishing a diagnosis of babesiosis in wild cervids. Post-mortem studies, PCR for identification of *B. odocoilei* 18S rDNA and sequencing have been used to confirm the diagnosis [20,29]. 

### 6.3. Epidemiology

As reviewed by Schoelkopf et al. [29], in the US, *B. odocoilei* is widely distributed and has been found affecting elk, reindeer, and caribou. In addition, *B. odocoilei* has also been identified in wild bovids such as the desert bighorn sheep (*Ovis canadensis nelsoni*), and captive musk oxen (*Ovibos moschatus*). The same authors reported a prevalence of 100%, 100%, and 12% in three elk farms from New Hampshire. In Canada, *B. odocoilei* was identified as the cause of mortality in captive reindeer and elk in the provinces of Quebec, Ontario, and Manitoba [20]. Recently, *B. odocoilei* was detected in 15 out of 21 ticks collected from dogs and cats, and in 4 out of 32 questing ticks collected in Southern Ontario [53,64] (Table 3).

## 7. Equine Piroplasmosis

Equine piroplasmosis (EP) in NA is caused by *Babesia caballi* and *Theileria equi* (former *B. equi*) [71]. Recently, a new species named *T. haneyi* was identified in horses at the US-Mexico border [21]. EP affects several equid species, but in NA, the disease has been documented only in horses [30,31]. Canada is considered free of EP. In the US, occasional outbreaks of EP have occurred, with the last one in 2009 [31]. Mexico, in contrast, is considered enzootic for EP [71]. Ixodid ticks in the genera *Dermacentor* and *Amblyomma* are vectors [18,31], but blood transfusion, mechanical and iatrogenic transmission are also possible [66]. 

**Figure 3:**
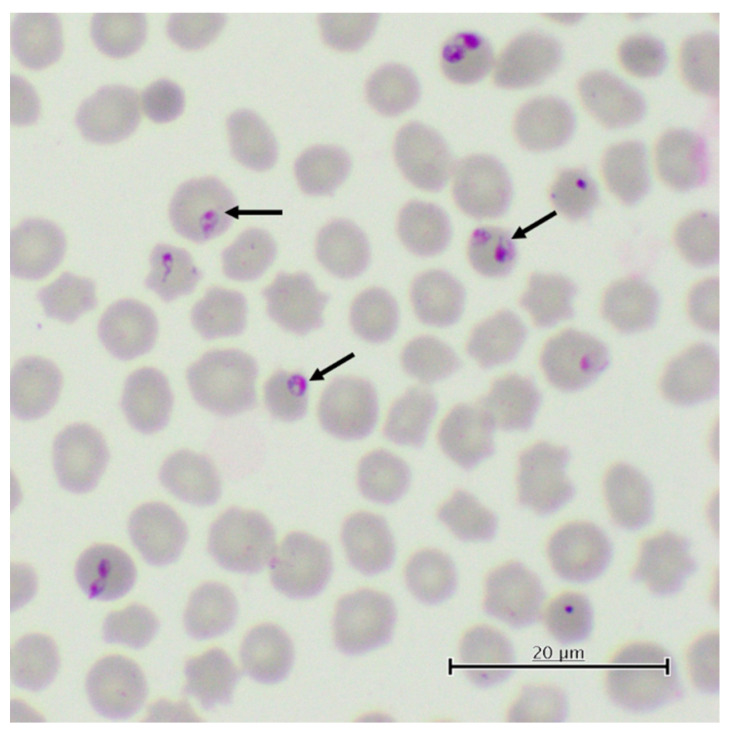
Giemsa-stained red cells containing intraerythrocytic inclusions of *Theileria equi* (arrows pointed). Merozoites may be pyriform, round, or ovoid, measuring 2–3 μm-long. They can occur singly, in pairs, or forming tetrads.

Merozoites of *B. caballi* are typically pear-shaped and large in size, measuring 2–5 μm in length and 1.3–3 µm in diameter (Figure 1B) [72]. Merozoites of *T. equi* may be pyriform, round, or ovoid measuring 2–3 μm long (Figure 3) [73]. In *T. equi*, intraerythrocytic inclusions forming tetrads known as “Maltese cross” may be found in blood smear preparations [73,74]. Individual masses of the tetrads measure 1.9 ± 0.2 µm in length and 0.9 ± 0.1 µm in width. For *T. haneyi*, the intraerythrocytic forms are ring or pyriform in shape and the Maltese cross may be seen. However, individual masses of the *T. haneyi* tetrads are smaller than *T. equi*, measuring 1.2 ± 0.2 µm in length and 0.7 ± 0.1 µm in width [21]. 

### 7.1. Pathogenesis

The life cycles of *T. equi* and *B. caballi* differ in that *B. caballi* sporozoites, transmitted by tick saliva, infect erythrocytes directly, while *T. equi* sporozoites infect lymphocytes and monocytes, where schizogony takes place, before infecting erythrocytes [18,71]. In both cases, replication of merozoites results in the rupture of host cells. Pathologies of the two piroplasms are similar and include hemolytic anemia, hemoglobinuria, and icterus. Infection of *T. equi* is more severe than *B. caballi* [71]. Significant clinical signs other than a mild fever were not observed in horses experimentally infected with *T. haneyi* [75]. The prepatent period is 12 to 19 days for *T. equi*, 7 to 60 days for *T. haneyi* [75], and 10 to 30 days for *B. caballi* [72,74]. 

EP is classified as hyperacute, acute, subacute or chronic [74]. The hyperacute form is characterized by a sudden onset of clinical signs that can lead to death. In acute infections, horses show inappetence, fever, anemia, hemoglobinuria or bilirubinuria, peripheral edema, and sometimes colic or diarrhea [76], contrasting with the chronic form, when symptoms are usually nonspecific such as inappetence, poor performance, and weight loss [76,77]. Some EP-infected mares can abort or give birth to stillborn foals after intrauterine infection [78]. Hematological changes include a decrease in packed cell volume (PCV), hemoglobin, red blood cells, and sometimes thrombocytes [79,80,81]. Horses, including foals infected in utero, become chronic carriers with a risk of transmitting the parasite to areas where the disease is not present [76,82,83]. Young horses seem to be more affected by *T. equi* infection than old ones [71]. Horses that recover from the acute infection become persistently infected and are reservoirs for about 4 years [71,78]. Gross lesions include subcutaneous edema, splenomegaly, hepatomegaly, enlarged kidneys, and endocardial hemorrhages [18,73]. 

### 7.2. Diagnosis

Clinical signs consistent with EP along with the detection of piroplasms on stained blood smears during acute EP are useful for clinical diagnosis [74]. However parasitemia can be low, complicating light microscopic examination of blood smears [84,85]. Serological techniques for detection of EP include ELISA, immunochromatographic test (ICT), IFAT, Western blot and complement fixation test (CFT). CFTs are the serologic method of choice to confirm EP [86,87]. Serologic tests vary in sensitivity and their uses are limited during prepatent infections. For chronic infections, IFAT or ELISA are recommended, with IFAT being able to differentiate between *T. equi* and *B. caballi*. PCR-based techniques have been widely used [73,88,89] for detecting piroplasms. Other diagnostic methods include loop-mediated isothermal amplification (LAMP) and in vitro culturing, cell cultures being useful to detect piroplasms in low-parasitemia blood samples [73,85]. The EP diagnostic recommendations of the World Organization for Animal Health (OIE) are to combine serological tests and PCR to determine if animals are free from infection, and microscopy and PCR to confirm clinical cases. 

### 7.3. Epidemiology

*B. caballi* was first introduced in the US in 1959 through horses imported from Cuba and spread through the southeastern states where the tick vector, *D. nitens*, was established [90]. The first case of *T. equi* was detected in 1964 [18] and through the eradication efforts of the USDA, the US was declared free of EP in 1988 [72]. Outbreaks of EP have occurred in Florida in 2008 [30] and in south Texas in 2009 [30]. In Texas, 292 infected horses were confirmed by ELISA and infections were associated with transmission by *Amblyomma mixtum* (former *A. cajennense*) (Table 4) [31]. Other reports of EP in the US have consisted of sporadic cases in several states, involving racehorses linked to unsanctioned racing, and horses imported to the US before implementation of the CFT in 2005, when it became the official import test [31,72].

In Mexico, EP has been present for several years, with the isolation of both *B. caballi* and *T. equi* reported in 1972 [19]. Currently, Mexico is considered prevalent for *B. caballi* and endemic for *T. equi* [62,63,89]. *B. caballi* and other small piroplasms have been morphologically identified in the southeastern state of Yucatan [52]. IFAT showed a prevalence of 27.4% of antibodies to *B. caballi* (Table 3) [63], whereas a report from northeastern Mexico reported a prevalence of 2.8% determined by nested PCR [62]. Reports of the prevalence of *T. equi* include 6%, 18%, and 19% in the North-Central, Gulf, and Western regions, respectively, using PCR [63,88,89]. A seroprevalence of 61.7% in Northeastern Mexico, using IFAT, has been reported [63] (Table 4).

## 8. Theileriosis in Cattle 

Theileriosis is caused by *Theileria* spp., which affects ruminants, equids, woodrats, and foxes [98,99]. Several species have been described and categorized as host-cell transforming and non-transforming species. Among the transforming species are *T. parva* and *T. annulata* in cattle, water buffalo (*Bubalus bubalis*), and yaks *(Bos grunniens)* that cause East Coast fever and Tropical theileriosis respectively. *T. parva* and *T. annulata* do not occur in NA. In the US, *T. orientalis*, genotype buffeli has been historically reported and is typically considered non-pathogenic (Table 1) [91,100]. However, in 2017, *T. orientalis*, genotype ikeda was reported in a cow-calf beef herd in Virginia and is now considered emerging [22]. Other *Theileria* non-transforming species include *T. mutans*, *T. velifera*, and *T. cervi*, with only *T. cervi* being present in NA. *T. cervi* typically results in sub-clinical infection, with only a few reports of clinical disease in WTD, elk, and mule deer [23,94,95,96]. *T. cervi* is transmitted by *A. americanum*, the lone star tick. Reports of the presence of *T. cervi* are common in wild populations of WTD and other ungulates from south-central states of the US [32,33,97] and Northern Mexico [33].

### 8.1. Pathogenesis 

**Figure 4:**
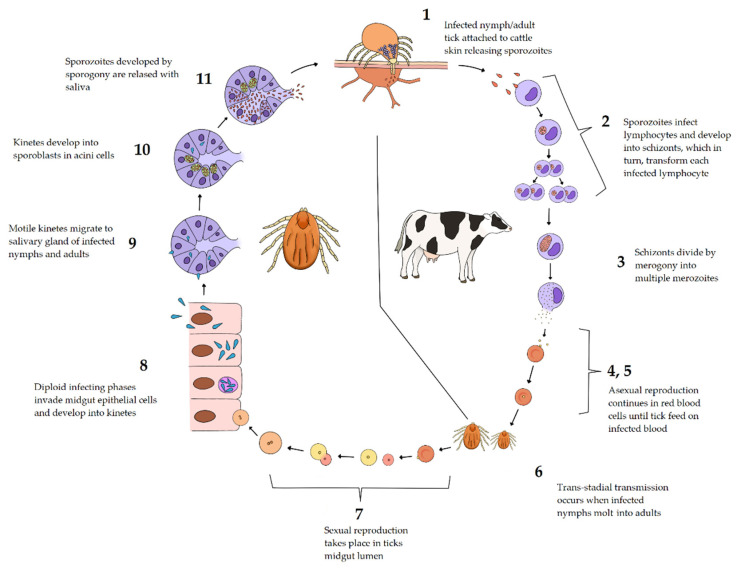
Life cycle of *Theileria*. 1 Infected nymph or adult ticks attach to skin, releasing sporozoites into host capillaries; 2 in *T. parva*, each sporozoite invades a lymphocyte and develops into a schizont. Schizonts transform infected lymphocytes inducing mitosis. Infected daughter cells are generated, doubling the number of infected lymphocytes; 3 merogony occurs and multiple merozoites are generated from each schizont; 4,5 merozoites are released into the bloodstream and invade erythrocytes to form the “piroplasm” stage and undergo asexual reproduction; 6 piroplasms are taken by nymphs and adult ticks during blood feeding; transstadial transmission occurs when infected nymphs molt into adults. 7 sexual reproduction occurs when micro and macro gametes, released from erythrocytes in the intestinal lumen, fused and form a zygote; 8 the zygote penetrates intestinal cells, developing into kinetes; 9 motile kinetes travel through hemolymph, infecting several tissues including salivary gland type III acini; 10 kinetes develop into sporoblasts; 11 sporogony takes place in salivary glands, where thousands of sporozoites are produced and released with tick saliva. Image created with PaintTool SAI, SYSTEMAX 1996–2022.

The life cycle of *Theileria* spp. Initiates when a tick ingests a blood meal from the vertebrate host containing piroplasms. These undergo syngamy in the tick gut and spread through the hemolymph to the tick salivary glands, where sporogony and formation of infective sporozoites occur. After the tick bite, the sporozoites contained in the tick saliva invade host leukocytes, where schizonts form. After schizogony, merozoites are released into the bloodstream, and in turn invade erythrocytes, producing more merozoites and trophozoites [101,102]. During infection with *T. orientalis*, the intraerythrocytic form is the major pathogenic stage, for *T. parva* it is the intralymphocytic form, and for *T. annulata* and *T. equi*, both forms are considered pathogenic [74,103]. *Theileria* spp. are transmitted transstadially within tick vectors, requiring a 2- or 3-host tick species [104] (Figure 4). 

Infection with *T. orientalis* is usually subclinical or results in mild disease [105]. Nevertheless, in recent years, it has been linked to sporadic outbreaks causing clinical signs and significant losses [106,107]. Mild infections of *T. orientalis* usually lead to anemia and hypoxia due to erythrocyte destruction, while severe disease can cause pyrexia, weakness, increased heart and respiratory rates, and sometimes abortion [108,109,110]. *T. orientalis* is grouped into 11 genotypes: types 1–8 and N1–N3. Types 1, 2 and 3 are also referred to as chitose, ikeda and buffeli respectively. Among these genotypes, only chitose and ikeda are pathogenic [111]. Most clinical cases caused by *T. orientalis* are associated with stress and immunosuppression in hosts [112].

### 8.2. Diagnosis

A combination of clinical signs along with serological and molecular methods help the diagnosis of *T. orientalis* infections. In the US, clinical signs of *T. orientalis* in cows from Virginia were weakness, icterus and anemia [22]. *T. orientalis* parasitemia is usually low, and detection in the blood is rare and unspecific [113]. Serological detection methods include ELISA, IFAT, and latex agglutination, which target piroplasms [111]. DNA-based molecular methods are also available and helpful for differentiating between genotypes [111] 

### 8.3. Epidemiology 

The first cases of bovine theileriosis in the US were reported in 1950 in Kansas and Texas [92], with the first identified as *T. mutans* and the second as *T.* *orientalis*, genotype buffeli. Reports of *T. orientalis* in the US include individual cases caused by the genotype buffeli in 1999 and 2000 in cows from Missouri and North Carolina [24,91], genotype buffeli cows in Michigan in 2002 [93], and genotype ikeda in Virginia, 2017 [22]. The report of *T. orientalis* from Virginia included cows from three separate herds. Infection of *T. orientalis* genotype ikeda was detected in 10 animals at two different times. Ages of infected cows ranged from 3 months to 13 years (Table 4). Three of these animals were clinically ill with icterus, low packed cell volume (PCV), and parasitemia. Infections of *T. orientalis*, genotype ikeda on these three Virginia farms were associated with infestations by *H. longicornis*, the longhorned tick, on cattle. *H. longicornis* is native to Asia, but has been introduced to the US and is now present in several eastern and central states [22]. The origin of the source of *T. orientalis*, genotype ikeda in Virginia is not clear. 

## 9. Conclusions

The current manuscript aimed to review the pathogenesis, diagnosis, and epidemiology of babesiosis and theileriosis disease in humans, domestic and wild animals in NA. Based on the reviewed literature, human babesiosis caused by *B. microti* is a problem in the Northeastern and Midwestern US, where the number of annual cases show an increasing trend due to the geographical expansion of the tick vector, *I. scapularis.* As result of this expansion, new cases have been detected in Canada and the Southern US. In Mexico, although a few cases of human babesiosis have been reported, tick vectors and wild animal reservoirs have yet to be identified. The small amount of information on human babesiosis and other tick-borne pathogens in Mexico is due to the lack of serological and molecular diagnostic methods, which are not yet widely available to researchers in the country. 

In NA, domestic animals are impacted by several species of tick-borne Apicomplexa. Several species of large and small *Babesia* continue to threaten the health of domestic dogs. Bovine babesiosis limits the movement of animals from infested areas, affecting exportation of cattle from Mexico to the US, where restrictions on importation of animals have been implemented to prevent re-establishment of the tick vector, *R. microplus*. The recent detection of cattle theileriosis, linked to the presence of the longhorned tick *H. longicornis*, outbreaks of equine piroplamsosis in the US, and the newly discovered species, *T. haneyi* are just a few examples of the complexity, challenges and research opportunities surrounding ticks and tick-borne pathogens in US, Canada, and Mexico.

## Figures and Tables

**Table 1:** Common or clinically relevant *Babesia* and *Theileria* species affecting humans and animals in North America.

Pathogen	Tick Vectors	Affected Hosts	References
*Babesia microti*	*Ixodes scapularis*	Humans	[7,8,9]
*B. duncani*	*Dermacentor albipictus*	Humans	[10]
*B. divergens*-like	ND	Humans	[11]
*B. bigemina*	*Rhipicephalus microplus*, *R. annulatus*	Cattle	[12,13,14]
*B. bovis*	*R. microplus*, *R. annulatus*	Cattle	[12,13,14]
*B. gibsoni*	*R. sanguineus* *	Dogs	[15]
*B. vogeli*	*R. sanguineus*	Dogs	[15]
*B. conradae*	ND	Dogs	[16]
*B. vulpes*	ND	Dogs	[17]
*B. caballi*	*D. albipictus*, *D. variabilis*, *D. nitens*	Equids	[18,19]
*B. odocoilei*	*I. scapularis*	White-tailed deer, reindeer, and elk	[20]
*Theileria equi*	*D. nitens*; *Amblyomma mixtum*	Horses	[18]
*T. haneyi*	ND	Horses	[21]
*T. orientalis* genotype ikeda	*Haemaphysalis longicornis*	Cattle	[22]
*T. cervi*	*A. americanum*	White-tailed deer, elk, and mule deer	[23,24]

ND, Not determined; * Transmission of *B. gibsoni* by *R. sanguineus* has not been demonstrated in NA.

**Table 2:** Relevant reports and surveys of human babesiosis in North America.

Species	Samples	Number of Confirmed Cases	Year	Confirmation Method	Geographical Location	References
*B. microti*	Blood, serum	7612 cases	2011–2015	Microscopy, PCR, Animal inoculation, and IFAT	New York, US * Massachusetts, US Connecticut, US New Jersey, US Rhode Island, US Wisconsin, US Minnesota, US	[40]
*B. microti*	Blood, serum	14,159	2011–2018	Microscopy, PCR, Animal inoculation, and IFAT	New York, US * Massachusetts, US Connecticut, US New Jersey, US Rhode Island, US Wisconsin, US New York City, US Maine, US Minnesota, US	[6]
*B. microti*	Blood	4	2015	PCR for 18S rRNA	Yucatan, MX	[25]
*B. microti*	Blood	1	2013	PCR for 18S rRNA	Manitoba; Can	[4]
*B. duncani*	Serum, blood	14	1966–2009	IFAT and PCR for ITS	California, US Washington, US Oregon, US	[10]

* From highest to lowest prevalence.

**Table 3:** Reports and surveys of *Babesia* spp. in domestic and wild animals in North America.

Species	Sample/Host	Host	Method	Prevalence (%)	Location	References
*B. bovis*	Serum	Cattle	IFAT	50	Nuevo Leon, MX	[50]
	Blood	Cattle	Microscopy	2.78	Yucatan, MX	[52]
	Serum	Cattle	Indirect ELISA	73.8	Yucatan, MX	[49]
	Cow Serum	Cattle	IFAT	98	Veracruz, MX	[51]
	Serum	Water buffalo	IFAT	71.4	Veracruz, MX	[51]
	Blood	Cattle	Nested PCR for CYTb	82.3	Veracruz, MX	[51]
	Blood	Water buffalo	Nested PCR for CYTb	16.2	Veracruz, MX	[51]
	Blood	WTD	Nested PCR	1.7	Northeast	[53]
	Blood	WTD	IFAT	16 & 4	La Salle and Webb counties, TX, US	[54]
	Blood	WTD	PCR for 18S rDNA	12	Tom Green Co, TX, US	[55]
	Serum	WTD	IFAT	59.9	Northeast, MX	[53]
	Blood	Nilgai	PCR for Rap-1	5	Coahuila, MX	[56]
	Blood	Fallow deer	PCR for Rap-1	9.5	Tamaulipas, MX	[57]
*B. bigemina*	Serum	Cattle	IFAT	56	Nuevo Leon, MX	[50]
	Blood	Cattle	Microscopy	1.2	Yucatan, MX	[52]
	Serum	Water buffalo	IFAT	85	Veracruz, MX	[51]
	Serum	Cattle	IFAT	100	Veracruz, MX	[51]
	Blood	Cattle	Nested PCR for CYTb	94.1	Veracruz, MX	[51]
	Blood	Water buffalo	Nested PCR for CYTb	24	Veracruz, MX	[51]
	Blood	Cattle	Microscopy	2.5	Yucatan, MX	[52]
	Serum	Cattle	IFAT	57	Southeast MX	[48]
	Blood	Nilgai	PCR for Rap-1	25	Coahuila, MX	[56]
	Blood	WTD	Nested PCR	4.2	Northeast, MX	[53]
	Serum	WTD	IFAT	5.4	Northeast, MX	[53]
	Blood	Fallow deer	PCR for Rap-1	4.7	Tamaulipas, MX	[57]
*B. vogeli*	Blood	Dogs	Microscopy	NA, Detection	Veracruz, MX *	[44]
	Blood	Dogs	PCR for 18S rRNA	6.6	Morelos, MX	[58]
	*R. sanguineus*	Dogs	PCR 18S rRNA	5.5	Morelos, MX	[58]
	Blood	Dogs	Microscopy	3.9	Yucatan, MX *	[52]
	Blood	Dogs	PCR	6.9	29 states, US	[59]
	Blood	Dogs	PCR for 18S rRNA	0.31 & 1.7	North Carolina	[17]
*B.gibsoni*	Blood	Dogs	PCR	91	29 states, US, and Ontario, CA	[59]
*B. conradae*	Blood	Dogs	Microscopy and PCR for 18S rRNA and ITS-2	NA (Isolation and detection)	California, US	[16]
	Blood	Dogs	PCR for 18S rRNA and ITS-2 region	52.7	South-central California, US	[28]
	Blood	Dogs	18S rRNA	37.5	Oklahoma, US	[60]
*B. rossi*	Blood	Dogs	PCR for 18S rRNA	NA Only detection	Texas, US	[61]
*B. vulpes*	Blood	Dogs	PCR for 18S rRNA	0.20	North Carolina	[17]
*B. caballi*	Blood	Horses	Microscopy	NA	Veracruz, MX	[19]
	Blood	Horses	PCR, for BC48	20.8	Juarez, MX	[62]
	*Otobius megnini*	Horses	PCR	5.9	Juarez, MX	[62]
	Sera	Horses	IFAT	27.4	Nuevo Leon, MX	[63]
*B. odocoilei*	Blood	White-tailed deer	Microscopy	2	Texas	[32]
	Blood	Reindeer and elk	PCR	-	Quebec, Ontario, and Manitoba, CA	[20]
	*Ixodes scapularis*	Dogs, cats, and questing ticks	PCR	71 & 12.5	Southern Ontario, CA	[64]
	Blood	Free-ranging desert bighorn sheep (*Ovis canadensis nelsoni*)	Microscopy and PCR for SSU rRNA gene	-	California, US	[29]
	Blood	Captive musk oxen (*Ovibos moschatus*)	Microscopy and PCR for SSU rRNA gene	2 cases	Minnessota, US	[29]
	Sera	Elk	IFAT	100, 100, & 26 in 3 farms	New Hampshire, US	[29]
	Blood	Elk	PCR	Detection of one case	New Hampshire, US	[29]
	Blood	Reindeer	PCR	Detection of three cases	Pennsylvania and New York, US	[29]

* Reported as *B. canis.*

**Table 4:** Reports and surveys of *Theileria* spp. in domestic and wild animals in North America.

Species	Sample	Host	Method	Prevalence (%)	Location	References
*T. equi*	blood	Horse	Microscopy and		Veracruz, MX	[19]
			Nested PCR for EMA-1	6.9		
		Soft ticks	Nested PCR for EMA-1	5.9	Juarez, MX	[62]
	Blood		Nested PCR for EMA-1	19.7	Jalisco, MX	[88]
	Blood	Horse	Microscopy	3.79	Yucatan, MX	[52]
	Serum	Horse	IFAT	45.2	Nuevo Leon, MX	[63]
	Serum	Horse	CFT, IFAT, and cELISA	9.5	Florida, US	[30]
	serum	Horse	c-ELISA	81.1	Southern Texas, US	[31]
*T. haneyi*	blood	Horse	PCR for 18S rDNA and nPCR for EMA	First report	Southern Texas, US	[21]
*T. orientalis* genotype ikeda	blood	Cattle (beef cattle)	PCR for SSU rRNA	Detection in 3 beef cattle	Virginia, US	[22]
*T.orientalis* genotype buffeli	blood	Cattle (beef cattle)	Microscopy IFAT	39 35	Missouri, US (1 herd)	[91]
	blood	Cattle (Angus)	PCR for SSU rRNA	Detected in a cow	North Carolina, US	[92]
	Blood and ticks	Cattle (mixed breed cow)	PCR for SSU rRNA	Detection in 3 cows and *A.americanun* and *D. variabilis* ticks	Missouri, US	[24]
	blood	Cattle (beef cattle)	PCR for SSU rRNA	Detection	Michigan, US	[93]
*T. cervi*	Blood	White-tailed deer *	Microscopy	72	Texas, US	[32]
	Blood	White-tailed deer	Microscopy	57	Texas, US	[94]
	Blood	Free-ranging elk	PCR for 18S rRNA	Case report	Canada **	[95]
	Blood	Free-ranging elk	PCR for 18S rRNA	Case report	Oklahoma, US	[95]
	Blood	White-tailed deer	PCR for 18S rRNA	Case report	Texas, Oklahoma, and Missouri, US	[95,96]
	Blood, liver, lymph nodes, and spleen	Mule deer	Microscopy and PCR for 18S sRNA	Case report	Oklahoma, US	[23]
	Blood	White-tailed deer	PCR for 18S rRNA	97.6 in Wild animals 40.4 in farmed animals	Florida, US	[97]
	Blood	White-tailed deer	Microscopy and PCR for 18S rRNA	Detection in 3 animals	Northeastern, MX	[33]

* First report; white tailed deer, ** Canadian Origin, but infected in Oklahoma, US.

## Data Availability

Not applicable.
